# Implementation of the Treat-to-Target Approach in Psoriatic Arthritis and Its Outcomes in Routine Clinical Practice

**DOI:** 10.7759/cureus.50507

**Published:** 2023-12-14

**Authors:** Shamas U Din, Muhammad Ahmed Saeed, Muhammad R Hameed, Maryam Aamer, Umbreen Arshad, Hafiz Yasir Qamar

**Affiliations:** 1 Rheumatology, Central Park Teaching Hospital, Central Park Medical College, Lahore, PAK; 2 Rheumatology, National Hospital and Medical Center, Lahore, PAK; 3 Rheumatology, Arthritis Care Foundation, Lahore, PAK

**Keywords:** disease-modifying anti-rheumatic drugs, clinical practice, dapsa, treat to target, psoriatic arthritis

## Abstract

Background

Measuring disease activity in psoriatic arthritis using validated tools and treating to a target (T2T) is advocated. It improves quality of life and delays radiographic progression. In clinical practice, it guides therapy escalation to achieve better disease control. This study aimed to assess the real-life implementation of the T2T concept in daily clinical practice and the proportion of patients achieving the target of low disease activity or remission.

Methodology

In this study, a retrospective review of patients diagnosed with psoriatic arthritis having clinical visits from January 2020 to February 2023 was done. The proportion of patients in whom disease activity was monitored using the Disease Activity Index for Psoriatic Arthritis (DAPSA) 28 and Physician Global Assessment (PGA) and those achieving the target was calculated using SPSS version 21 (IBM Corp., Armonk, NY, USA).

Results

A total of 89 patients were included in the study after fulfilling the inclusion and exclusion criteria. Overall, 56.2% (50) of patients were males and 43.8% (39) were females, with a mean age of 43.5 ± 14.5 years, mean disease duration of 6.6 ± 3.8 years, and mean follow-up duration of 2.8 ± 1.6 years. Of the study population, 43.8% (39) had axial involvement, 23.6% (21) had dactylitis, and 12.4% (11) had enthesitis. Skin psoriasis was present in 84.3% (75), 11.2% (10) had a family history of psoriasis, 19.1% (17) had nail changes, 1.1% (1) had uveitis, and in 94.8% (73) of patients skin psoriasis presented before arthritis.

Overall, 97.7% (85) of patients were on conventional synthetic disease-modifying antirheumatic drugs (csDMARDs), with the most common being methotrexate in 77%, followed by leflunomide in 8%. Further, 34.8% (31) were using biological DMARDs (bDMARDs), with the most common being tofacitinib (33.7%), infliximab (28.1%), and secukinumab (24.7%) being other choices. Overall, 21.1% (18) of patients experienced adverse events with csDMARDs and 3.2% (1) with biological DMARDs. DAPSA28 was recorded in 44.9% (40), Psoriasis Area and Severity Index in 16.8% (15), and PGA in 100% of patients. Target of low disease activity (LDA)/remission was achieved in 50.6% (45) patients, as assessed by PGA or DAPSA28 cutoff. The LDA/remission target was achieved in 51.2% of patients taking csDMARDs, and 74.2% in those who were on bDMARDs.

Conclusions

It is crucial to measure the disease activity using validated tools and treat the patient to target for achieving better disease control and improved quality of life. Despite the evidence that T2T improves outcomes, it is not widely practiced in routine clinical practice.

## Introduction

Psoriatic arthritis (PsA) belongs to a group of rheumatic diseases and seronegative spondylo-arthropathies that have common genetic associations and share clinical features aside from peripheral arthritis, such as spondylitis, enthesitis, dactylitis, uveitis and inflammatory bowel disease [1]. It can affect up to 30% of patients with psoriasis over their lifetime [2]. Psoriatic skin patches and nail disease are the cutaneous symptoms of PsA. It is associated with increased morbidity resulting from joint damage over time, leading to poor health-related quality of life and functional capacity compared to healthy populations. Maintaining sustained minimal disease activity is crucial in PsA, as it is associated with low progression of radiologic joint damage over time [3]. Hence, a treat-to-target (T2T) strategy is employed in the management of PsA. Historically, there were limited options for the management of PsA, but with the advent of effective therapies including biologic therapies, it is now possible to achieve minimal disease activity.

The benefits of a T2T approach for PsA were first revealed in the TICOPA (Tight Control of Inflammation in Early Psoriatic Arthritis) trial [4]. Treating to target necessitates defining a quantifiable target; in PsA, this target is recognized as remission or low disease activity (LDA) both by the international T2T task force and by the updated European League Against Rheumatism treatment recommendations [5]. Although the Group for Research and Assessment of Psoriasis and Psoriatic Arthritis (GRAPPA) recommendations do not specifically recommend a treatment target, they agree that the ultimate goal of therapy is to achieve the lowest disease activity [6]. In a retrospective study that included patients in a PsA registry during 2016-2017, 117 records were analyzed, and it was found that the T2T approach was implemented in 76 (65.5%) patients [7].

The objective of this study was to assess the implementation of the T2T concept in daily clinical practice using Physician Global Assessment (PGA) of disease activity or formal composite disease activity measures using the Disease Activity Index for Psoriatic Arthritis (DAPSA) 28 or clinical DAPSA28. The primary outcome was the proportion of patients in whom the target approach was used with measurement of disease activity and those achieving remission and/or LDA of PsA as assessed by DAPSA28. The potential remission targets were DAPSA28 or clinical DAPSA28 remission (≤4) for PsA.

## Materials and methods

The study was conducted at the Department of Rheumatology/Institute of Rheumatic Diseases, Central Park Teaching Hospital, Lahore, and its affiliated clinics. After obtaining approval from the institutional review board (approval number: CPMC/IRB-No/1393, dated 20-03-2023), the patients presenting during the study period, i.e., from January 2020 to February 2023, were enrolled in the study according to the inclusion/exclusion criteria. Electronic records from these centers were used and data were collected using a questionnaire. A retrospective analysis of the electronic records of patients was done. The sample size for this study was all patients presenting within the study period who fulfilled the inclusion and exclusion criteria. The sampling technique used was non-probability consecutive sampling. All patients visiting the hospital consented that their data could be used for research purposes without revealing their identity.

Inclusion and exclusion criteria

Patients aged 18 years or more, of either gender, fulfilling the Classification Criteria for Psoriatic Arthritis (CASPAR) criteria, and having at least two visits were included. Patients having concurrent inflammatory diseases were excluded from the study.

PsA was defined according to the CASPAR criteria which consist of confirmed inflammatory articular disease (joint, spine, or enthesis) fulfilling at least three of the following features: current psoriasis (assigned a score of 2 points; all other features are assigned a score of 1), a history of psoriasis or a family history of psoriasis (unless current psoriasis is present), dactylitis, juxta-articular new bone formation (hands or feet), rheumatoid factor negativity (except latex test), and psoriatic nail dystrophy [8].

DAPSA28 was calculated using the following formula: [TJC + SJC + patient global VAS (cm) + pain VAS (cm) + C-reactive protein (CRP; mg/L)]. Clinical DAPSA28 was calculated using the following formula: (TJC +SJC + patient global VAS + pain VAS). The following formula is used to calculate the Psoriasis Area and Severity Index (PASI) score: PASI = 0.1 (Eh + lh + Sh) Ah + 0.2 (Eu + lu + Su) Au + 0.3 (Et +lt + St) At + 0.4 (El +ll +Sl) Al [9].

DAPSA remission was labeled when DAPSA28 or clinical DASPA28 score was ≤4, and DAPSA LDA was labeled when DAPSA28 or clinical DASPA28 score was ≤14.

Data analysis

The data were analyzed using SPSS version 21 (IBM Corp., Armonk, NY, USA). Categorical variables were described as frequencies and percentages. Quantitative variables were described as mean and standard deviation. The outcome variables were stratified with the use of disease-modifying antirheumatic drugs (DMARDs) and biological therapies. The chi-square test was used at a 5% level of significance to determine differences using DMARDs and biological therapies. Data are presented in the form of tables and figures.

## Results

A total of 89 patients were included in the study after fulfilling the inclusion and exclusion criteria. Overall, 56.2% (50) of patients were males and 43.8% (39) were females, with a mean age of 43.5 ± 14.5 years, a mean disease duration of 6.6 ± 3.8 years, and a mean follow-up duration of 2.8 ± 1.6 years. Further, 43.8% (39) had axial involvement, 23.6% (21) had dactylitis, and 12.4% (11) had enthesitis. Skin psoriasis was present in 84.3% (75), 11.2% (10) had a family history of psoriasis, 19.1% (17) had nail changes, 1.1% (1) had uveitis, and in 94.8% (73) patients, skin psoriasis presented before arthritis (Table 1).

**Table 1:** Demographics and disease characteristics. csDMARDs: conventional synthetic disease-modifying anti-rheumatic drugs; DAPSA: Disease Activity in Psoriatic Arthritis

Variable	% or mean
Age (years)	43.5 ± 14.5
Disease duration (years)	6.6 ± 3.8
Follow-up duration (years)	2.8 ± 1.6
Males	56.2% (n = 50)
Females	43.8% (n = 39)
Polyarthritis	97.8% (n = 87)
Oligoarthritis	2.2% (n = 2)
Axial involvement	34.8% (n = 39)
Dactylitis	23.6% (n = 21)
Enthesitis	12.4% (n = 11)
Skin psoriasis	84.3% (n = 75)
Nail psoriasis	19.1% (n = 17)
Uveitis	1.1% (n = 1)
csDMARDs use	97.8% (n = 87)
Biologics use	43.8% (n = 31)
DAPSA at baseline	18.3 ± 11.2
DAPSA at follow-up	9.4 ± 6

**Figure 1:**
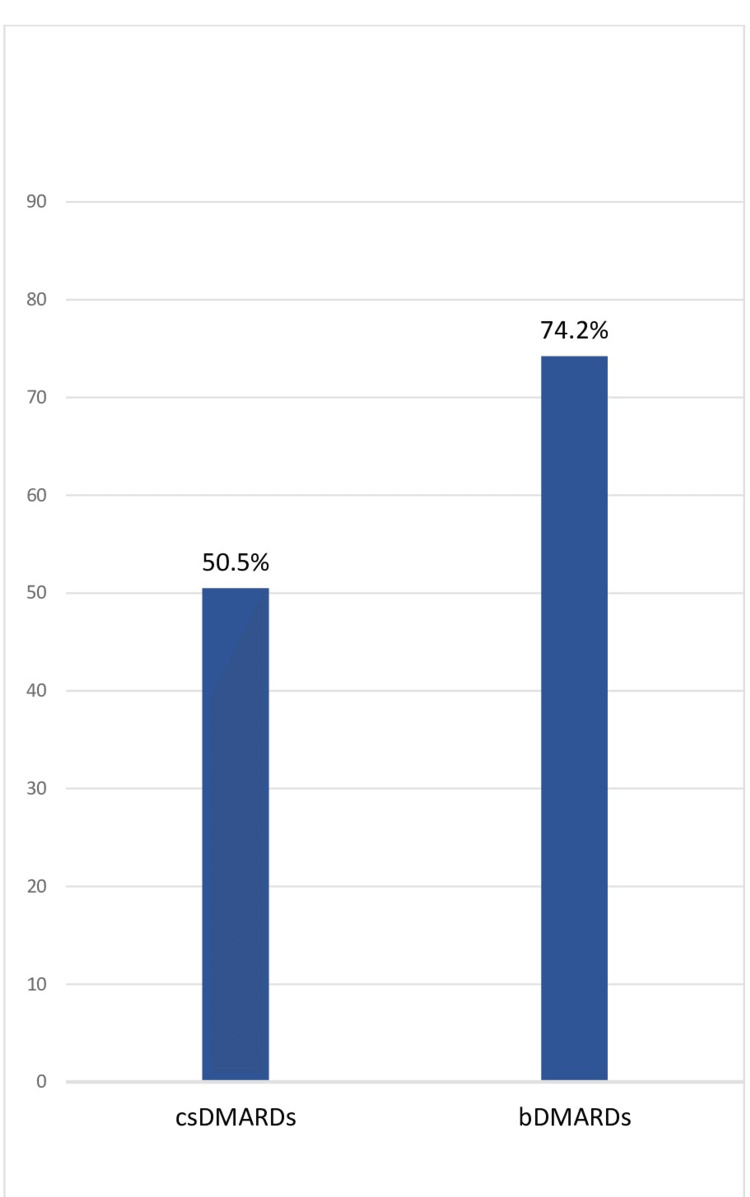
Comparison of remission/low disease activity achieved. csDMARDs: conventional synthetic disease-modifying anti-rheumatic drugs; bDMARDs: biologic disease-modifying anti-rheumatic drugs

**Figure 2:**
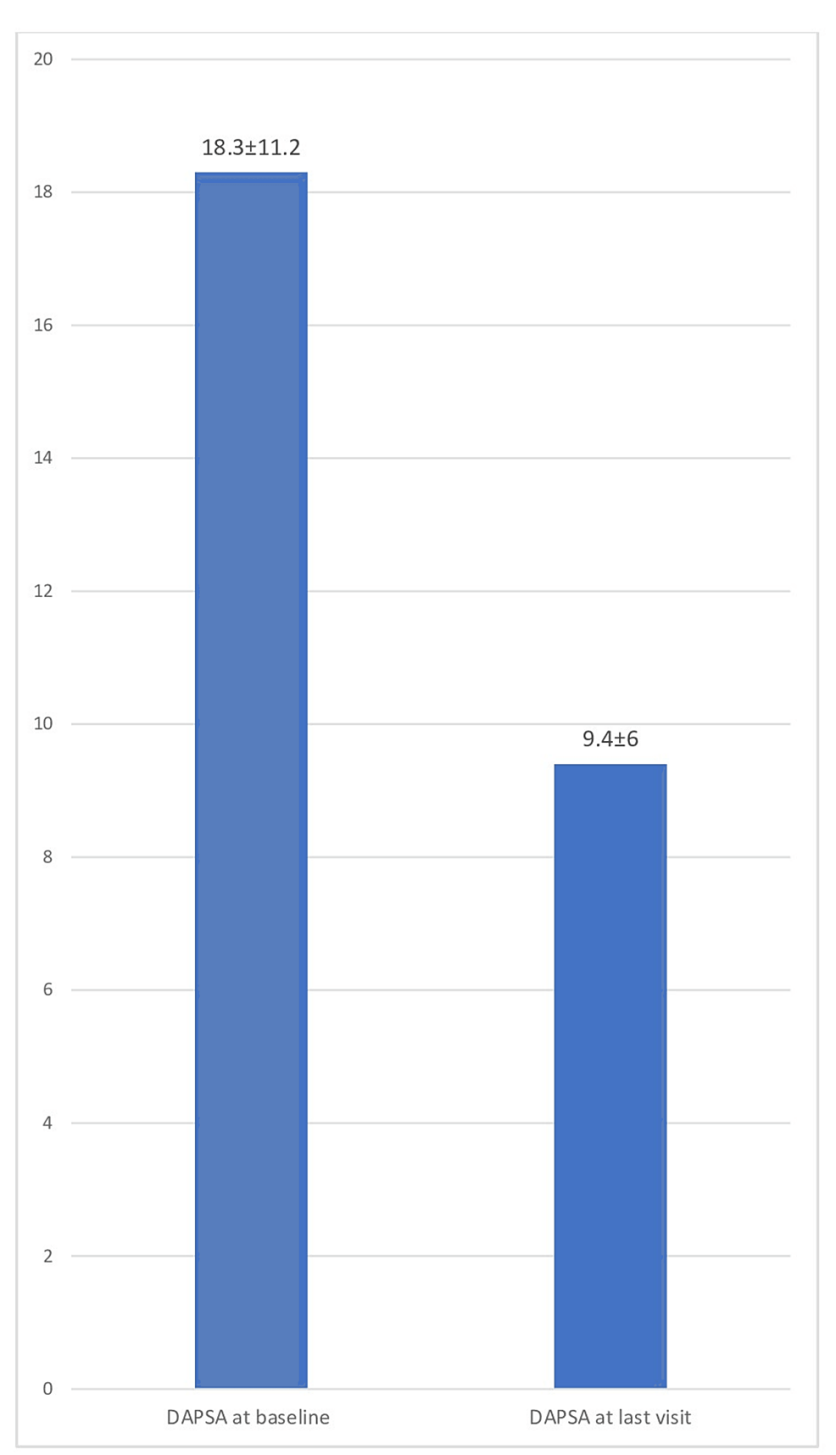
Comparison of DAPSA at the baseline and the last follow-up visit. DAPSA: Disease Activity in Psoriatic Arthritis

Of the study population, 97.7% (85) of patients were on conventional synthetic DMARDs (csDMARDs), with the most common being methotrexate in 77%, followed by leflunomide in 8%. Overall, 34.8% (31) were using biological DMARDs (bDMARDs), of which the most common were tofacitinib (33.7%), infliximab (28.1%), and secukinumab (24.7%). Further, 21.1% (18) of patients experienced adverse events with csDMARDs and 3.2% (1) with bDMARDs. DAPSA28 was recorded in 44.9% (40) and PGA in 100% of patients. The LDA/remission target was achieved in 50.6% (45) patients, as assessed by the PGA or DAPSA28 cutoff. The LDA/remission target was achieved in 51.2% of patients taking csDMARDs and in 74.2% taking bDMARDs (Figure 1). DAPSA at baseline was 18.3 ± 11.2 and 9.4 ± 6 at the last follow-up visit (Figure 2).

## Discussion

This is the first-ever study from Pakistan examining the use of a T2T approach in PsA in clinical practice. The T2T approach in PsA patients was studied initially in the TICOPA trial, which showed that tight control of disease activity improves joint outcomes in PsA without a significant increase in unexpected adverse events [4]. In our study, disease activity was recorded in 44.9% utilizing DAPSA28, while PGA was recorded in 100% of patients. In another study, the T2T strategy was employed in 65.5% of patients. The major hurdles in the implementation of the T2T approach were the limited time for a complete clinical assessment involving many disease domains and the unavailability of simple, reliable, and composite disease activity measures that include all disease domains [1]. Psoriatic disease is complex, comprising multiple clinical manifestations, which significantly impact patients’ function and quality of life. There has been a new trend toward the development of disease activity measures that are responsive to clinical measures and patient-reported outcomes, thereby including the full burden of disease. Widespread implementation of these tools in routine clinical practice and research trials will be very useful in providing clinicians with a better understanding of the full patient experience, helping treatment decisions, and increasing patient satisfaction and efficacy of newer therapies in PsA [10].

Patients treated to a target using composite disease activity measures had less radiographic progression as observed in a randomized placebo-controlled study of golimumab therapy in PsA [11]. Our study was a retrospective analysis of electronic medical records and patients who were not followed for radiographic progression comparing the patients achieving LDA/remission with those who did not. In a study conducted in the Asia Pacific region, the majority of PsA patients received csDMARDs (81%) with a relatively low prevalence of bDMARDs (24%). Regarding T2T, 32% and 40% of patients with PsA achieved minimal disease activity (MDA) and DAPSA LDA, respectively [12].

In routine clinical practice, it is very challenging to calculate disease activity measures due to the short duration of clinical encounters. In these limited-duration routine clinic visits, which typically last 15-20 minutes, it is difficult to gather information for different disease activity measures and various components of MDA and document it [13]. In an international survey in 2017, it was found that 45% of physicians were using disease activity measures, with the most commonly used being MDA [14]. In our study, disease activity measures in the forms of DAPSA28 were calculated in 44.9% of patients but PGA was recorded in all patients.

Our study has limitations in that it was a retrospective analysis of 89 patients and did not include an assessment of radiographic progression. Further studies are needed in our population to assess the impact of the T2T approach utilizing formal composite disease activity measures in routine clinical practice in prospective cohorts and determine radiographic progression comparing those who achieved remission/LDA to those who did not. It highlights the importance of special clinics for PsA patients. These clinics will help develop the expertise of physicians and other staff to assess and manage patients with PsA. In other diseases, special clinics for complex diseases have allowed thorough and systematic assessment of patients resulting in better management and improved quality of care [15].

## Conclusions

Measuring the disease activity using validated tools and treating the patient to target for achieving better disease control and improved quality of life are crucial. Despite the evidence that T2T improves outcomes, it is not widely applied in routine clinical practice. This study highlights the need to implement it in clinical practice to improve patient outcomes.
